# Primary Pulmonary Epithelioid Hemangioendothelioma: A Rare Cause of PET-Negative Pulmonary Nodules

**DOI:** 10.1155/2011/262674

**Published:** 2011-08-22

**Authors:** Riccardo Cazzuffi, Nunzio Calia, Franco Ravenna, Claudio Pasquini, Sara Saturni, Giorgio Narciso Cavallesco, Francesco Quarantotto, Rosa Rinaldi, Annaluisa Cogo, Gaetano Caramori, Alberto Papi

**Affiliations:** ^1^Sezione di Malattie dell'Apparato Respiratorio, Dipartimento di Medicina Clinica e Sperimentale, Università di Ferrara, Italy; ^2^Dipartimento di Scienze Chirurgiche, Anestesiologiche e Radiologiche, Modulo di Chirurgia Toracica, Università di Ferrara, Italy; ^3^Sezione di Anatomia, Istologia e Citologia Patologica, Dipartimento di Medicina Sperimentale e Diagnostica, Università di Ferrara, Italy; ^4^Centro per lo Studio delle Malattie Infiammatorie Croniche delle Vie Aeree e Patologie Fumo Correlate dell'Apparato Respiratorio (CEMICEF; formerly Centro di Ricerca su Asma e BPCO), Università di Ferrara, Via Savonarola 9, 44121 Ferrara, Italy

## Abstract

We report here a case of primary pulmonary epithelioid hemangioendothelioma diagnosed in a 67-year-old Caucasian man, presenting with exertion dyspnoea, dry cough, and multiple bilateral pulmonary nodules revealed by computed tomography. At the 18F-fluorodeoxyglucose positron emission tomography, these nodules were negative. The histopathological diagnosis was made on a pulmonary wedge resection (performed during video-thoracoscopic surgery).

## 1. Introduction

Pulmonary epithelioid hemangioendothelioma (PEH) is a rare, low- to intermediate-grade tumor of endothelial origin [1] with around 120 cases reported in the literature [2]. At the onset, the patients are usually asymptomatic or present with nonspecific symptoms (such as weight loss, fatigue, dyspnoea, cough, and chest pain). Chest imaging usually shows the presence of multiple, bilateral small pulmonary lesions. The diagnosis usually requires a lung biopsy. The treatment is still not standardized. Surgery is suggested in presence of a solitary nodule or few unilateral nodules. Instead if the lesions are unresectable or multiple and bilateral several chemotherapy protocols have been used. The prognosis is variable with a median survival of 4-5 years. 

## 2. Case Report

A 67-year-old Caucasian man, lifelong nonsmoker, with no prior history of lung diseases, was referred to our university hospital for the presence, in the last 3 months, of exertion dyspnoea (grade 1 according to the Medical Research Council scale [3]) and dry cough. He had worked as an employee without occupational exposures of clinical relevance. His past medical history was characterized by an herniated lumbar disc, systemic arterial hypertension (treated with calcium blockers), diabetes mellitus (treated with oral hypoglycaemic agents), and polycythemia vera (diagnosed in 2007 and treated from 2008 with hydroxyurea). Physical examination was unremarkable. Arterial blood gases analysis performed with the patient breathing room air demonstrated normal values (pH 7.40, PaCO_2_ 43 mmHg, PaO_2_ 91 mmHg, and bicarbonate level 26 mmoL/L). Routine laboratory tests were within the normal range except for those listed in Table 1. Serum levels of neoplastic markers were within normal range, including carcinoembryonic antigen (2.7 ng/mL), prostate-specific antigen (3.75 ng/mL), and CA19.9 (15 U/mL). Also the serum levels of angiotensin-converting enzyme (11 IU/mL) and *β*_2_ microglobulin (2.50 mg/mL) were within normal limits. 

**Figure 1:**
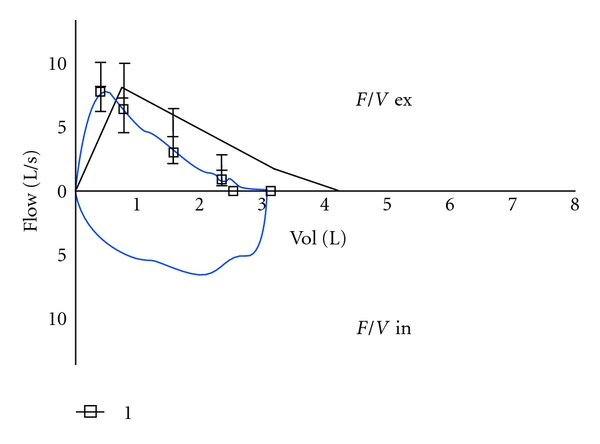
Flow volume loop.

The pulmonary function tests showed the presence of a restrictive pattern with a vital capacity (VC) of 3.14 litres (70% of the predicted value), a forced vital capacity (FVC) of 3.11 litres (72% of the predicted value), forced expiratory volume in one second (FEV_1_) of 2.55 litres (76.5% of predicted value), a FEV_1_/VC ratio of 81% (108% of the predicted value) and a total lung capacity (TLC), 5.35 litres (73.3% of the predicted value). DLCO was in the normal range (Figure 1). 

**Figure 2:**
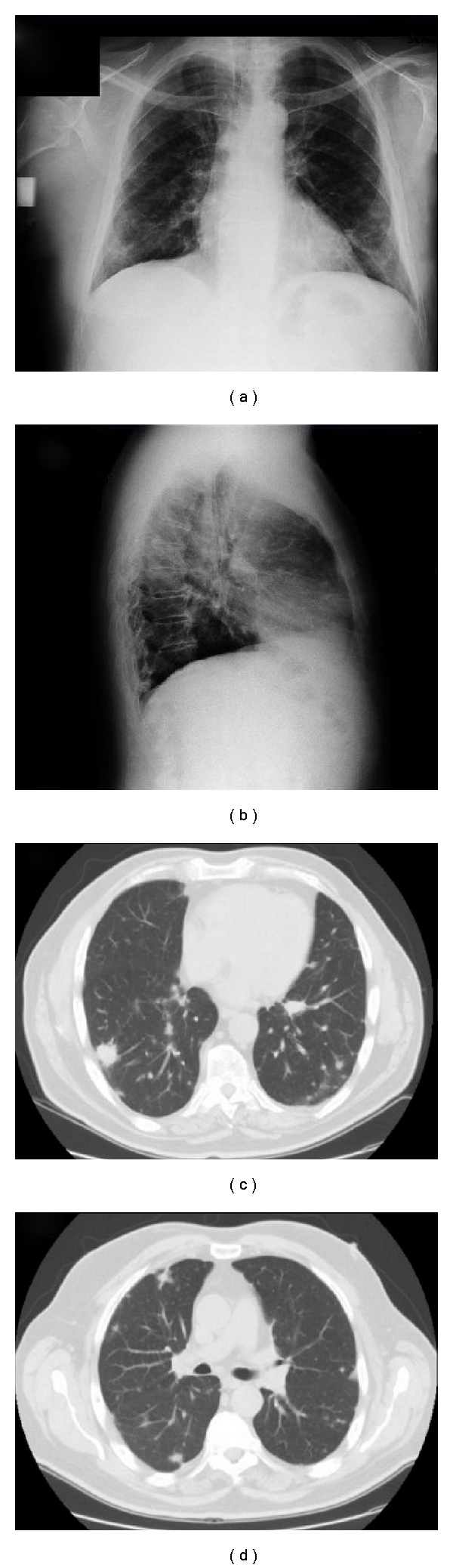
The chest radiography (a, b) and a computed tomography of the chest (performed with iodine intravenous contrast medium) (c, d) showed the presence of multiple bilateral pulmonary nodules.

The chest radiography and a computed tomography of the chest (performed with iodine intravenous contrast medium) showed the presence of multiple bilateral pulmonary nodules (with a diameter variable between 3 mm and 3 cm) without contrast enhancement and the presence of one nodule (with the diameter of 2 cm) in the fourth hepatic segment showing late-phase contrast enhancement and another in the spleen (with the diameter of 1 cm) with cystic features (Figure 2). 

**Figure 3:**
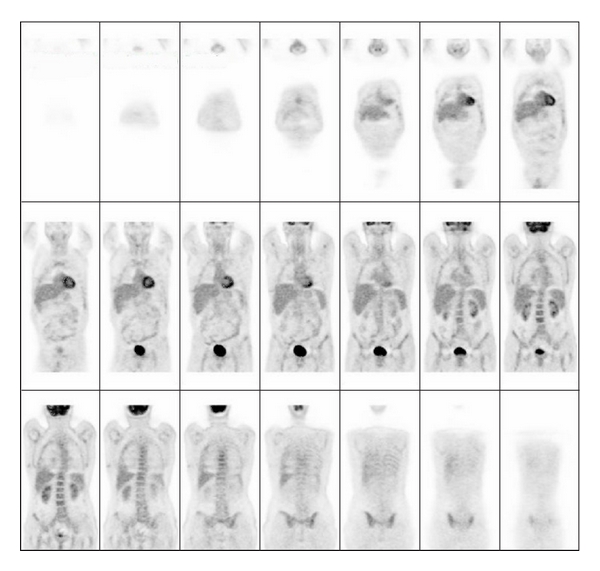
18F-fluorodeoxyglucose positron emission tomography, all these nodules were not showing any uptake of the tracer.

Interestingly, at the 18F-fluorodeoxyglucose positron emission tomography (PET), all these nodules were not showing any uptake of the tracer (Figure 3). 

The fiberoptic bronchoscopy was normal. A transbronchial lung biopsy and an ultrasound-guided transthoracic lung biopsy were both nondiagnostic. 

The final diagnosis of pulmonary epithelioid hemangioendothelioma was made by pulmonary wedge resection of a nodule located in the left lower lobe by video-thoracoscopic surgery. Histopathological examination of the resected tissue revealed round- to oval-shaped nodules, with a central sclerotic, hypocellular zone, and peripheral zone rich of cells. There was tumour diffusion into the adjacent bronchioles and the alveolar spaces with micropolypoid aspects. The extracellular stroma consisted of an abundant matrix of chondroid, hyaline, mucinous, or myxomatous appearance. 

The neoplastic cells were of polygonal shape and eosinophilic with round nuclei and uniform small to moderately sized nucleoli. Some cells had cytoplasmic vacuoles. 

**Figure 4:**
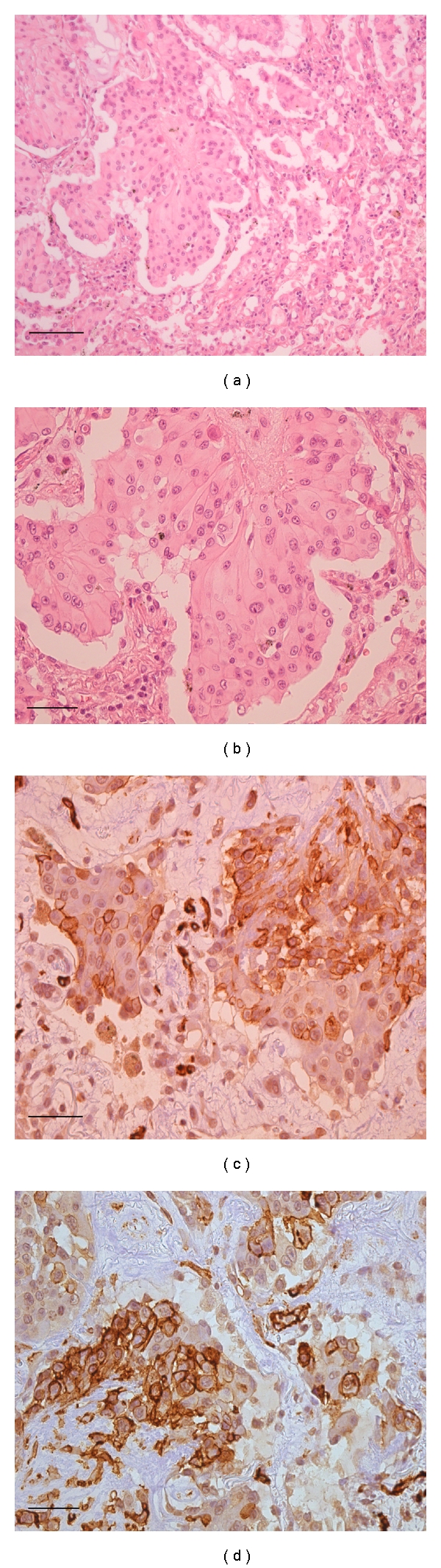
(a) Neoplastic nodule showing increased number of cells at the periphery with an eosinophilic stroma (H/E, 100x magnification). (b) The neoplastic cells are of polygonal shape and eosinophilic with round nuclei and uniform small to moderately sized nucleoli. (H/E, 200x magnification). (c) Immunoperoxidase staining for CD34 of the neoplastic cells (brown colour) (200x magnification). (d) Immunoperoxidase staining for CD31 of the neoplastic cells (brown colour) (200x magnification).

Immunohistochemical studies demonstrated that the neoplastic cells are endothelial cells immunoreactive for Von Willebrand factor, CD31, CD34, and vimentin (Figure 4). 

Immunostaining for other markers (including caudal type homeobox transcription factor 2 (CDX2), cytokeratin (CK) 20, CK7, prostate-specific antigen (PSA), pan-cytokeratin, chromogranin, epithelial membrane antigen (EMA), HMB45, progesterone receptor, and thyroid transcription factor (TTF)-1) was negative.

## 3. Discussion

Pulmonary epithelioid hemangioendothelioma (PEH) is a rare, low- to intermediate-grade tumor of endothelial origin [1]. Epithelioid hemangioendothelioma can arise from many organs, including lungs, liver, bone, and soft tissue, simultaneously or sequentially. When this occurs, it may be difficult to determinate if the tumor is multicentric from the beginning or there is a primary lesion with metastases to the other tissues [4]. Around 120 cases have been reported in literature [2, Table 2], only five of them were in Italian patients [5, 15–18]. Mean (SD) age of patients is 40.1 (17.5) years, and 73% are females [6]. Interestingly, our patient is instead a 67-year-old male. Most patients are asymptomatic at presentation, and some are complaining of weight loss, fatigue, dyspnoea, fever, pleuritic chest pain, mild nonproductive cough, and haemoptysis [19]. In most cases, the physical examination is normal, but few cases of digital clubbing and pleural effusions have been reported [1, 6]. 

The most characteristic feature of PEH on chest radiographs or CT is the presence of multiple perivascular nodules with well- or ill-defined margins in both lungs. The nodules may range in size up to 2 cm, but most of them are ≤1 cm in diameter; they are usually found near medium-sized vessels and bronchi [1]. This radiological presentation is suggestive of many lung diseases (Table 3). 

Some authors consider 18F-fluorodeoxyglucose positron emission tomography an important tool for PEH diagnosis [7]; however, in our patient, the PET was negative, suggesting that a negative PET cannot exclude PEH. Unfortunately, we do not have a molecular explanation for this finding but may be related to a low proliferation rate of the neoplastic cells.

The diagnosis of PEH is made on the basis of the histopathological features and confirmed using immunohistochemical staining for endothelial cell markers, such as against factor VIII-related von Willebrand antigen, CD31 or CD34 [2, 4, 13, 20, 21]. 

The prognosis is variable. The 5-year survival is around 60% (range 47–71%). In fact, there are two groups of PEH at the clinical presentation: (1) one asymptomatic, with a solitary pulmonary nodule or unilateral multiple nodules. Usually they can be managed with surgery alone, and lymphatic invasion is unlikely. Their prognosis is good with a median survival of more than 10 years; (2) another symptomatic group presents with multiple bilateral pulmonary nodules or pleural effusion with scarce response to chemotherapy. The prognosis of this group is poor [11]. Most patients die from pulmonary insufficiency as a result of an increasing number of tumor nodules [9]. Hematogeneous metastases are rare and have been described especially in the liver but also in other sites [2, 4].

The treatment of PEH is not standardized. Surgery alone is indicated in the presence of a single pulmonary nodule or unilateral multiple nodules. Lung transplantation should be considered in patients with vascular infiltration [10].

Various chemotherapies have been reported for unresectable or metastatic PEH, with variable effectiveness [22]. Other authors have suggested a role for the hormonal therapy (antiestrogens and progesterone) in patients with diffuse disease if the neoplastic cells express estrogen and progesterone receptors [23]. A slight regression of the pulmonary lesions was also obtained with interferon (IFN)-2*α*, probably for its antiangiogenic activity [24]. Thalidomide and IFN-*α* have also been proposed for unresectable cases [22]. Some beneficial results were also obtained with bevacizumab, a monoclonal antibody that blocks human VEGF-A [22, 25]. The neoplastic cells express glucocorticoid receptors and the enzyme 11*β*-hydroxysteroid dehydrogenase involved in the synthesis of the steroids, suggesting a potential role for steroid modulators [26]. 

In conclusion, we had the opportunity to observe an unusual primary pulmonary hemangioepithelioma in an old male patient, and interestingly, in comparison with the published literature, the neoplastic lesions were PET negative. Again, as previously suggested [27], we stress the importance of starting an international clinical registry of this unusual neoplasm.

## Figures and Tables

**Table 1:** Laboratory tests concerning diabetes mellitus and polycythemia vera.

White blood cells	12.01 × 10^3^	Normal range 4.00–11 × 10^3^/*μ*L
Red blood cells	4.80 × 10^6^	Normal range 4.50–6.50 × 10^6^/*μ*L
HGB	15.9	Normal range 13.0–18 g/dl
HCT	49	Normal range 40–54%
MCV	103	Normal range 76–96 fl
MCH	33.1	Normal range 27.0–32.0 pg/dl
MCHC	32.3	Normal range 30.0–35.0 g/dl
PLT	434	Normal range 150–450 × 10^3^/*μ*L
Blood neutrophils	9.56 × 10^3^	Normal range 2.00–7.50 × 10^3^/*μ*L
Blood lymphocytes	1.92 × 10^3^	Normal range 1.50–5.00 × 10^3^/*μ*L
Blood monocytes	0.43 × 10^3^	Normal range 0.20–1.00 × 10^3^/*μ*L
Blood eosinophils	0.08 × 10^3^	Normal range 0.04–0.40 × 10^3^/*μ*L
Blood basophils	0.01 × 10^3^	/
PT	1.09	Normal range 0.85–1.20 INR
APTT	1.22	Normal range 0.85–1.20 ratio
Glycaemia	99	Normal range 70–11 mg/dl
Creatinine	1.2	Normal range 0.9–1.4 mg/dl

APTT: Activated thromboplastin time. HCT: Hematocrit. HGB: Haemoglobin. LDH: Lactate dehydrogenase. MCH: Mean corpuscular Haemoglobin. MCV: Mean blood cell volume. MCHC: Mean cell haemoglobin concentration. PLT: Platelets. PT: Prothrombin time.

**Table 2:** Review of the clinical, radiological and pathological features of the published cases of primary pulmonary epithelioid hemangioendothelioma.

	Review of the literature	Our case report
Female/male ratio	3 : 1 female	Male

Mean (SD) age	40.1 (17.5) years	67 years

Symptoms	Weight loss, fatigue, and respiratory symptoms (dyspnoea, chest pain, mild nonproductive cough, and mild haemoptysis)	Dyspnoea and dry cough

Chest radiography and computed tomography	Usually multiple bilateral pulmonary nodules	Multiple bilateral pulmonary nodules

PET	Positive	Negative

Metastatic sites	Lymph nodes, liver, bone, skin, serosal membranes, spleen, tonsils, retroperitoneum, kidney and central nervous system	Single spleen and liver metastatic nodules

Immunohistochemical features	Factor VIII von Willebrand factor+, CD31+, or CD34+	Factor VIII von Willebrand factor+, CD31+, CD34+

5-year survival (%)	60%	Alive for eight months then lost at the follow up

Obtained with the data from [4–18].

**Table 3:** Differential diagnosis of multiple pulmonary bilateral nodules.

Metastases
Primary lung cancer (particularly bronchioloalveolar carcinoma)
Lymphoid tumors and myeloma
Leukaemic infiltrates
Benign vascular tumors (hemangioma and lymphangioma)
Malignant vascular tumors (angiosarcoma and Kaposi's sarcoma)
Neuroendocrine tumourlets
Nodular lesions in pulmonary fibrosis
Pneumoconiosis
Infections (tuberculosis, nocardiosis, aspergillosis, and histoplasmosis)
Sarcoidosis
Langerhan's cell histiocytosis
Vasculitis
Connectivitis
Pulmonary arteriovenous malformations

Obtained with the data from [4, 5, 9, 12–14].
